# Statistical Power Calculations for Mixed Pharmacokinetic Study Designs Using a Population Approach

**DOI:** 10.1208/s12248-014-9641-4

**Published:** 2014-07-11

**Authors:** Frank Kloprogge, Julie A. Simpson, Nicholas P. J. Day, Nicholas J. White, Joel Tarning

**Affiliations:** 1 Mahidol-Oxford Tropical Medicine Research Unit, Faculty of Tropical Medicine, Mahidol University, 420/6 Rajvithi Road, Bangkok, 10400 Thailand; 2 Centre for Tropical Medicine, Nuffield Department of Medicine, University of Oxford, Oxford, UK; 3 Centre for Epidemiology and Biostatistics, Melbourne School of Population and Global Health, The University of Melbourne, Melbourne, Australia

**Keywords:** mixed pharmacokinetic study designs, Monte Carlo Mapped Power, optimal pharmacokinetic study design, statistical power calculations

## Abstract

Simultaneous modelling of dense and sparse pharmacokinetic data is possible with a population approach. To determine the number of individuals required to detect the effect of a covariate, simulation-based power calculation methodologies can be employed. The Monte Carlo Mapped Power method (a simulation-based power calculation methodology using the likelihood ratio test) was extended in the current study to perform sample size calculations for mixed pharmacokinetic studies (*i.e.* both sparse and dense data collection). A workflow guiding an easy and straightforward pharmacokinetic study design, considering also the cost-effectiveness of alternative study designs, was used in this analysis. Initially, data were simulated for a hypothetical drug and then for the anti-malarial drug, dihydroartemisinin. Two datasets (sampling design A: dense; sampling design B: sparse) were simulated using a pharmacokinetic model that included a binary covariate effect and subsequently re-estimated using (1) the same model and (2) a model not including the covariate effect in NONMEM 7.2. Power calculations were performed for varying numbers of patients with sampling designs A and B. Study designs with statistical power >80% were selected and further evaluated for cost-effectiveness. The simulation studies of the hypothetical drug and the anti-malarial drug dihydroartemisinin demonstrated that the simulation-based power calculation methodology, based on the Monte Carlo Mapped Power method, can be utilised to evaluate and determine the sample size of mixed (part sparsely and part densely sampled) study designs. The developed method can contribute to the design of robust and efficient pharmacokinetic studies.

## INTRODUCTION

Population pharmacokinetic analyses can be performed on datasets consisting of densely, sparsely or a combination of densely and sparsely sampled data (1). The random variability in a population pharmacokinetic analysis is explained preferably in part by physiological covariates (*e.g.* body weight, age, sex and/or disease status) which are independent of the sampling framework being analysed. However, both the sampling schedule and the number of individuals studied affect the statistical power for hypothesis testing of these covariates. A dataset consisting of mixed sparsely and densely sampled individuals requires a different sample size for hypothesis testing compared to a study consisting of solely dense or sparse sampling. Furthermore, the ratio of sparsely to densely sampled individuals in a combined study design alters the precision of the parameter estimates. The impact of (combined) sampling schemes on the statistical power to detect covariate effects and the precision of the pharmacokinetic parameters should therefore be evaluated prospectively during the design phase of a study. This will ensure that the design is sufficient to answer the key research questions of the population pharmacokinetic study. In the current study, the Monte Carlo Mapped Power calculation method (2) (a simulation-based power calculation methodology using the likelihood ratio test) was adapted and developed further to enable the determination of statistical power for varying sample sizes of sparse only, dense only and mixed sparse/dense sampling study designs.

An example where statistical power calculations for mixed study designs are commonly required is a pharmacokinetic study nested within a clinical efficacy study. Typically, these studies include a relatively densely sampled pharmacokinetic study arm, implemented for a descriptive pharmacokinetic analysis, and a larger relatively sparsely sampled efficacy arm, which may have been implemented to evaluate the correlation between pharmacokinetic and pharmacodynamic endpoints. For example, in anti-malarial drug studies, correlations are sought between measures such as the day 7 concentrations and/or concentrations at the time of the recrudescent infection and cure rate (*e.g.* NCT00495508 at http://www.clinicaltrials.gov/). Optimal design methods have been used to ensure that the selected sampling designs are adequate for the descriptive and quantitative pharmacokinetic (-pharmacodynamic) analyses (*i.e.* sufficient precision for parameter estimates) (3–5). However, these types of pharmacokinetic studies are rarely designed incorporating the assumption that hypothesis testing of covariate effects can be performed using the sparsely and densely sampled study arms simultaneously. Often no sample size calculations are performed to detect the covariate effects on the full study population, yet assessing the two designs together may change the sample sizes required to detect the covariate effects. The impact of sampling design and number of individuals in mixed study designs should therefore be evaluated prospectively during the design phase of the study to avoid enrolling too few or too many patients.

Another situation where power calculations for mixed study designs could be useful is pooling of individual datasets for a meta-analysis. Power calculations for mixed study designs can confirm if the sample size available for the meta-analysis is sufficient to detect a clinically important effect of the covariate of interest. The calculated statistical power may highlight the need for inclusion of more studies to the pooled dataset and thereby save unnecessary work, as pooled population pharmacokinetic analyses are computationally intensive.

The aim of this study was to adapt and develop further the Monte Carlo Mapped Power method to perform statistical power calculations for mixed (sparsely and densely sampled) study designs. A workflow which guides an easy and straightforward pharmacokinetic study design, considering the cost efficiency of alternative study designs, was constructed and used during this study.

## METHODS

**Fig. 1:**
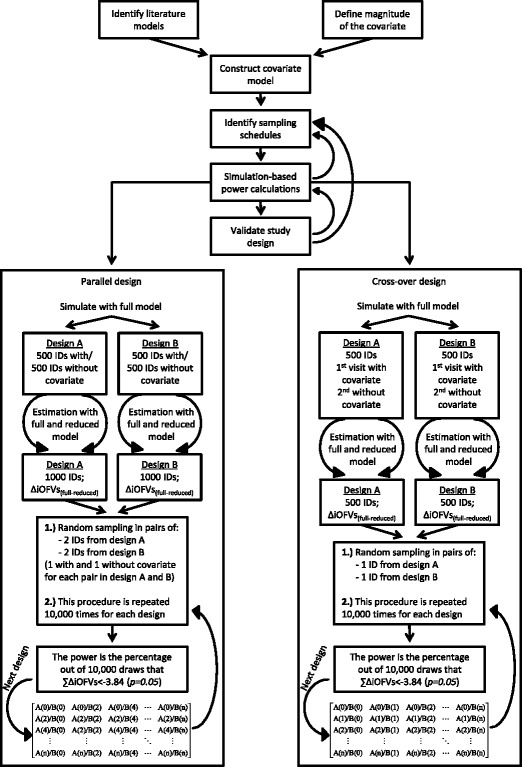
Visual representation of the applied workflow and power calculation methodology. *Identify literature models*: reference models from the literature were collected for the paradigm drugs; *define magnitude of the covariate*: the magnitude of covariate effect that resulted in clinically relevant differences in drug exposure was set; *construct covariate model*: a final covariate model to perform the simulations was constructed; *identify sampling schedules*: sampling schedules from the literature were identified (dihydroartemisinin example) or obtained using an optimal design methodology (hypothetical drug example); *simulation-based power calculation*: sample size calculation for the selected study designs and *validate study designs*: different study designs were assessed for expected parameter precision and expected cost. *A (N)* and *B (N)* in the depicted matrices represent the number of patients randomly drawn from dataset A (densely sampled patient in this study) and dataset B (sparsely sampled patients in this study) to create a new study design

The simulation-based statistical power calculation methodology for mixed pharmacokinetic study designs was assessed first through a predefined workflow (Fig. 1) on a hypothetical drug. The impact of different study designs (*i.e.* cross-over study design, parallel study design and different sampling schemes) on the required sample size to detect the covariate was evaluated. The findings of this simulation study were used subsequently to design a simulation study based on the pharmacokinetics of a currently available drug used to treat malaria. The anti-malarial, dihydroartemisinin, was used for this case study, and a pharmacokinetic study was designed to evaluate the effect of the binary covariate, pregnancy, on its pharmacokinetics.

### Pharmacokinetic Models

The pharmacokinetic model used for the hypothetical drug followed first-order absorption, one-compartment disposition pharmacokinetics and first-order elimination (Table I) (6). The model used for the dihydroartemisinin example followed transit-absorption, one-compartment disposition pharmacokinetics, first-order elimination and additional reported literature covariates (simulated according to the range of the reported study population) (Table I) (6). A binary covariate on elimination clearance was implemented in both the hypothetical drug (30% proportional increase) and the dihydroartemisinin (20% proportional increase) examples (Table I).

**Table I:** Summary of Simulation Assumptions for the Hypothetical Drug Example and the Dihydroartemisinin Example

	Hypothetical drug example		Dihydroartemisinin example	
Pharmacokinetic model				
	Fixed effects	IIV/IOV^†^	Fixed effects	IIV/IOV^†^
Transit compartments (n)	–	–	7	–
MTT (h)	–	–	0.982	0.23^†^
*k*_a_ (h − 1)	0.8	0.1	–	–
*F* (%)	–	–	100 (fixed)	0.0881
CL (L/h)	20	0.08/0.02^†^	78	–
*V* (L)	70	0.1	129	0.0162
Covariate on CL (%)	30	–	20	–
Residual error	10% (proportional)		0.58 (additive on LN data)	
Dataset				
	Parallel design	Cross-over design	Parallel design	
Sparse sampling times (h)	Window 1: 0, 0.5, 1, 1.5, 2, 2.5, 3 or 4 Window 2: 5, 6, 8, 10, 12, 16, 20 or 24	Window 1: 0, 0.5, 1, 1.5, 2, 2.5, 3 or 4 Window 2: 5, 6, 8, 10, 12, 16, 20 or 24	Pregnant window 1: 0.42–0.48, Window 2: 1.2–3.4, window3: 3.4–4.9 and Window 4: 6.0–8.0 Nonpregnant window1: 0.28–0.48, Window 2: 0.5–0.95, window3: 2.5–3.7 and Window 4: 5.8–6.6 (7)	
Dense sampling times (h)	0, 0.5, 1, 1.5, 2, 2.5, 3, 4, 5, 6, 8, 10, 12, 16, 20 and 24	0, 0.5, 1, 1.5, 2, 2.5, 3, 4, 5, 6, 8, 10, 12, 16, 20 and 24	0, 0.25, 0.5, 1, 2, 3, 4, 6, 8 and 12 (6)	
Number of patients	1,000	500	1,000	
Financial figures				
	Parallel design	Cross-over design	Parallel design	
Sparse sample hospital cost (units)	750	1,500	750	
Dense sample hospital cost (units)	1,000	2,000	1,000	
Price per analyses sample (units)	50	50	50	

*MTT* mean transit time, *k*_*a*_ absorption rate constant, *F* bioavailability, *CL* elimination clearance, *V* apparent volume of distribution, *IIV* inter-individual variability presented as variance, *IOV* inter-occasion variability presented as variance

^†^indicates that the estimate is IOV rather than IIV

### Statistical Power Calculation: Simulation and Estimation of Datasets

The simulation-based power calculation methodology for combined pharmacokinetic study designs employed in this study is based on the Monte Carlo Mapped Power method (2). First datasets (dense sampling: dataset A; sparse sampling: dataset B) were simulated and re-estimated. For the hypothetical drug example, a cross-over and parallel study design was evaluated. For the dihydroartemisinin example, only a parallel study design was assessed since the covariate of interest was pregnancy in malaria patients. The number of individuals in the pseudo-population has to be sufficient in order to obtain robust results from the statistical power calculations; for the parallel study design in this study a pseudo population of 1,000 individuals was simulated with 500 patients displaying and 500 patients not displaying the binary covariate. For the cross-over design, a single dataset of 500 individuals was simulated displaying the binary covariate during the first visit and not displaying the binary covariate during the second visit (Table I, Fig. 1). Simulations were executed using the population pharmacokinetic model containing the covariate effect of interest, inter-individual variability and inter-occasion variability, in NONMEM 7.2 (ICON Development Solutions, Ellicott City, MD, USA) on a Windows 7 operating system (Microsoft Corporation, Seattle, WA, USA) with a G-Fortran compiler (Free Software Foundation, Boston, MA, USA).

The blood/plasma sampling protocols (sparse and dense) were identical for the parallel and cross-over study designs in the hypothetical drug example (Table I). The sampling protocol for the hypothetical drug was obtained using PopED version 2.13 (pharmacometrics research group, Uppsala University, Sweden, http://poped.sourceforge.net/). This optimal sampling was also used for the sparse design where two samples per patient were drawn randomly from the selected sampling protocol (Table I). The sampling protocols for a dense and sparse study design for the dihydroartemisinin example were taken from literature (6,7). For patients in the sparsely sampled arm of the dihydroartemisinin example, a sample could be drawn at any time within the specified sampling windows (Table I).

The simulated datasets were re-estimated subsequently (first-order conditional estimation method with interaction) using (1) a model including the covariate effect (full model) and (2) a model not including the covariate effect (reduced model). Re-estimation of a simulated parallel design was performed without inter-occasion variability as only one occasion per individual was available.

### Statistical Power Calculations: Bootstrapping of New Design Combinations

At random, individuals (*N*) from dataset A and dataset B were drawn to create new design combinations in terms of number of patients with dense A (*N*) and sparse sampling B (*N*). This was performed until all possible combinations were assessed within the set boundaries (*i.e*. maximum number of patients with dense and sparse sampling). The difference in objective function value (OFV; calculated as minus twice the natural logarithm of the likelihood of the data) for the newly created dataset was obtained by deducting the OFV obtained with the full model from the OFV obtained with the reduced model. The number of times that the procedure was repeated for every design combination should be sufficient in order to get a robust sample size and in this study the procedure was repeated 10,000 times. The power was expressed as the percentage out of 10,000 draws that ΔOFV dropped more than 3.84 (*p* < 0.05) (Fig. 1). An R-package was developed (PharmPow, available on the Comprehensive R Archive Network (CRAN), http://cran.r-project.org/web/packages/PharmPow/PharmPow.pdf) and used for these calculations. A power of 80% in this study was considered to be the threshold, resulting in a statistically significant covariate in 80% of studies.

### Validation of Proposed Study Designs; Expected Parameter Precision

A selection of potential study designs (with at least 80% power) was evaluated further to determine the expected parameter precision. For each selected study design scenario, 1,000 datasets were simulated and re-estimated in order to obtain the expected relative standard errors on the estimates of the fixed and random effects, which include the between-subject, between-occasion and residual variability (Eq. 1).

1 $$ \mathrm{Expected}\ \mathrm{RSE}\left(\%\right)\kern0.5em =\kern0.5em 100\kern0.5em \times \kern0.5em \frac{\mathrm{standard}\ \mathrm{error}}{\mathrm{mean}\ \mathrm{parameter}\ \mathrm{estimate}} $$

### Validation of Proposed Study Designs; Financial Figures

The selection of study designs was also evaluated from a cost effectiveness standpoint (Eq. 2).

2 $$ \mathrm{Budget}\kern0.5em =\kern0.5em \mathrm{cost}\left(\mathrm{hospitalisation}\right)\times \mathrm{days}\kern0.5em (N)\kern0.5em +\kern0.5em \mathrm{cost}\left(\mathrm{assay}\right)\kern-21.2em \times \kern0.5em \mathrm{samples}\kern0.5em (N) $$

‘Days’ in Eq. 2 represent the sum of the total number of days that all patients were hospitalised during the study. ‘Samples’ in Eq. 2 represent the total number of samples analysed during the study.

The hospitalisation cost for patients in the densely sampled arm was chosen arbitrarily at 1,000 units. The hospitalisation cost for patients in the sparse sampled arm was chosen at 750 units to account for reduced enrolment time and staff costs. The cost of analysing one sample was set to an arbitrary value of 50 units.

## RESULTS

### Hypothetical Drug Example

**Fig. 2:**
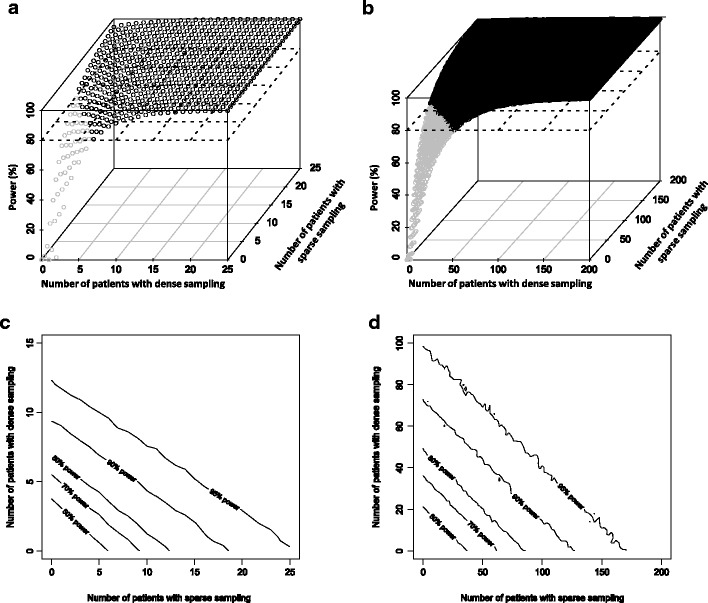
Statistical power for different study designs in a cross-over design (**a**, **c**) and a parallel design (**b**, **d**) for the hypothetical drug example using 3-D plots (**a**, **b**) and 2-D contour plots (**c**, **d**)

For a cross-over study design, 7 (dense sampling only), 9 (5 assigned to dense sampling/4 assigned to sparse sampling), 10 (3 assigned to dense sampling, 7 assigned to sparse sampling) or 13 (sparse sampling only) patients were required to detect a binary covariate (30% proportional increase in elimination clearance) with at least 80% statistical power (Fig. 2a, c). For a parallel study design (*i.e.* 50% of patients displaying the covariate and 50% of patients not displaying the covariate), a total of 52 (dense sampling only), 58 (34 assigned to dense sampling, 24 assigned to sparse sampling), 60 (18 assigned to dense sampling, 42 assigned to sparse sampling) or 66 (sparse sampling only) patients were required to detect a binary covariate (30% proportional increase in elimination clearance) with at least 80% power (Fig. 2b, d).

Precision of parameter estimates improved with an increased number of plasma concentrations samples (Table II). For example, the precision on *V* and *k*_a_ was improved by the inclusion of densely sampled patients compared to a study design consisting of solely sparsely sampled patients in both the parallel and cross-over settings (Table II). Furthermore, precision on all parameter estimates was better using a parallel study design compared to a cross-over study design (Table II).

**Table II:** Selected Summary Results of Different Study Designs in Terms of Parameter Precision and Study Costs for the Hypothetical Drug Case

Study design	Sampling design (*N*)			Samples (*N*)			Parameter precision (%RSE)									Study Cost	Patient cost
	Total	Dense ID’s	Sparse ID’s	Total	Dense	Sparse	CL	*V*	*k*_a_	cov	CL IIV	V IIV	*k*_a_ IIV	CL IOV	RUV	Hypothetical	Hypothetical
Cross-over	7	7	0	224	224	0	12.4	12.2	13.3	34.4	66.4	62.5	85.3	60.3	11.0	25,200	3,600
	9	5	4	176	160	16	11.5	13.3	16.9	34.3	68.3	68.8	106	68.9	12.5	24,800	2,760
	10	3	7	124	96	28	11.5	13.8	19.7	34.9	68.3	79.4	139	75.5	15.7	22,700	2,270
	13	0	13	52	0	52	11.5	19.0	53.6	37.4	69.4	114	222	106	32.8	22,100	1,700
Parallel	52	52	0	832	832	0	5.71	4.82	5.71	34.0	22.3	23.0	36.6	–	5.93	93,600	1,800
	58	34	24	592	544	48	5.90	5.35	6.74	35.8	24.3	27.7	45.1	–	6.96	81,600	1,410
	60	18	42	372	288	84	6.31	6.30	8.71	36.2	26.0	34.2	63.0	–	9.45	68,100	1,140
	66	0	66	132	0	132	6.83	14.0	22.9	39.5	33.6	62.8	128	–	32.7	56,100	850

The sampling design was expressed as total number of patients (*i.e.* number of patients in each occasion in the cross-over design, or 50% of patients displaying the categorical covariate and 50% of patients not displaying the categorical covariate in the parallel design). Parameter precision (relative standard error) was calculated as 100 × (standard error/mean parameter estimate) obtained from 1,000 simulated and re-estimated studies. The study cost was calculated as: cost (hospitalisation) × days (*N*) + cost (assay) × samples (*N*) using 1,000 units and 750 units as hospitalisation costs per day for dense and sparse designs, respectively, and an assay cost of 50 units per sample

*RSE* relative standard error, *ID* individuals, *CL* elimination clearance, *V* apparent volume of distribution, *k*_*a*_ absorption rate constant, *IOV* inter-occasion variability, *cov* covariate, *IIV* inter-individual variability, *RUV* residual unexplained variability

As expected, a cross-over study design is potentially less expensive to conduct compared to a parallel design since fewer patients and samples are needed to achieve 80% statistical power. Sparse sampling study designs are less expensive to conduct compared to dense sampling study designs in both the cross-over and parallel studies.

### Dihydroartemisinin Example

**Fig. 3:**
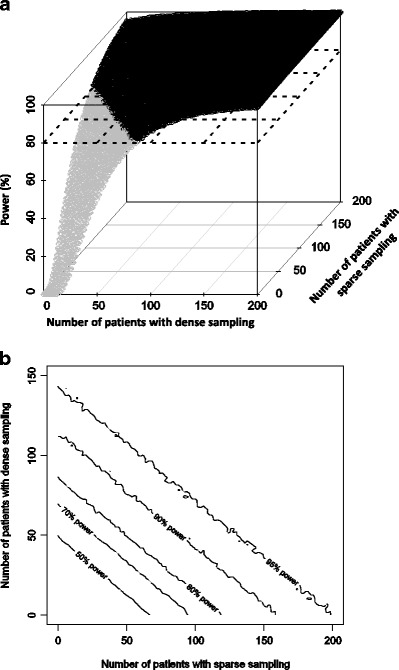
Statistical power for different study designs in a parallel design for the dihydroartemisinin example using a 3-D plot (**a**) and a 2-D contour plot (**b**)

Using a parallel study design, 88 (dense sampling only), 98 (56 assigned to dense sampling, 42 assigned to sparse sampling), 108 (28 assigned to dense sampling, 80 assigned to sparse sampling) or 120 (sparse sampling only) patients were required to detect the pregnancy effect (20% proportional increase in elimination clearance) with 80% power (Fig. 3a, b).

Between-subject variability on *V* and the residual error displayed substantial differences between the different study designs but values of the relative standard errors did not reach levels higher than 20% (Table III).

**Table III:** Selected Summary Results of Different Study Designs in Terms of Parameter Precision and Study Cost for the Dihydroartemisinin Case

Sampling design (*N*)			Samples (*N*)			Parameter precision (%RSE)								Study Cost	Patient cost
Total	Dense ID’s	Sparse ID’s	Total	Dense	Sparse	CL	*V*	MTT	PREG_CL	PARA_F	V_IIV	F_IIV	Sigma	Hypothetical	Hypothetical
88	88	0	880	880	0	4.97	5.39	0.98	16.5	5.65	21.9	23.4	2.68	132,000	1,500
98	56	42	728	560	168	5.05	5.80	1.16	18.1	5.70	25.8	25.5	3.05	124,000	1,260
108	28	80	600	280	320	5.11	6.12	1.39	18.8	5.94	31.5	26.4	3.54	118,000	1,090
120	0	120	480	0	480	5.45	7.06	1.79	21.4	5.55	43.4	27.7	4.42	114,000	950

The sampling design was expressed as total number of patients (*i.e.* 50% of patients displaying the categorical covariate and 50% of patients not displaying the categorical covariate). Parameter precision (relative standard error) was calculated as 100 × (standard error/mean parameter estimate) obtained from 1,000 simulated and re-estimated studies. The study cost was calculated as cost (hospitalisation) × days (*N*) + cost (assay) × samples (*N*) using 1,000 units and 750 units as hospitalisation cost per day for dense and sparse designs, respectively, and an assay cost of 50 units per sample

*RSE* relative standard error, *ID* individuals, *CL* elimination clearance, *V* apparent volume of distribution, *MTT* mean transit time, *PREG* pregnant, *IV* inter-individual, *PARA* parasitemia (i.e. additionally reported model covariate)

The more densely sampled patients were included in the study design, the more expensive the total study became (Table III).

## DISCUSSION

Optimal design methodologies and statistical power calculations contribute to robust and efficient pharmacokinetic study designs. Different power calculation methodologies can be employed for sample size calculations such as a simulation-based power calculation method or a power calculation method based on an analytical solution (*i.e.* optimal design approach) (8).

The Wald test (analytical solution using an optimal design approach) has traditionally been used for sample size calculations of parallel, cross-over and mixed pharmacokinetic study designs (8,9). However, a simulation-based approach can also be applied for all of these study designs (10). An advantage of a simulation-based method, compared to the Wald test, is that the same statistical methodology is used for the power calculations as for the actual pharmacometric analysis of the data (log-likelihood ratio test). A disadvantage of a simulation-based approach is that it is computationally intensive, in particular when models and/or study designs become more complex. The Monte Carlo Mapped Power method, which is a simulation-based sample size calculation method, has proven to provide robust and accurate sample size calculations with substantially reduced computation times (2). However, the Monte Carlo Mapped Power method has, to the best of our knowledge, not yet been applied to mixed pharmacokinetic study designs. The aim of the current study was to modify the simulation based Monte Carlo Mapped Power method, in order to enable sample size calculations for mixed pharmacokinetic study designs (2). The methodology was evaluated successfully using a workflow which also considered the cost efficiency of alternative study designs.

The Monte Carlo Mapped Power method relies heavily on the number of patients in the pseudo-dataset and the number of times that each new study design is sampled randomly from the pseudo-population. Vong and colleagues compared the Monte Carlo Mapped Power approach to the traditional computationally intensive stochastic simulation and re-estimation approach and demonstrated that the Monte Carlo Mapped Power method was able to provide robust and accurate power estimates at less than 1% of the run-time of a stochastic simulation and re-estimation approach (2).

### Dihydroartemisinin Example

The pregnancy effect on dihydroartemisinin elimination clearance was implemented as a categorical covariate (20% proportional increase) for simulation purposes. This has a smaller impact on dihydroartemisinin exposure compared to the 37.5% decrease in dihydroartemisinin bioavailability observed previously in a population pharmacokinetic study of data from 24 pregnant and 24 nonpregnant women with uncomplicated malaria (6). Thus, the proposed study design would therefore be sufficient to detect the 37.5% decrease in dihydroartemisinin bioavailability actually observed in the clinical setting.

### Cross-over *vs* Parallel Design

The hypothetical drug example indicated that a cross-over design requires substantially fewer patients and plasma concentration samples to detect statistically the covariate effect compared to a parallel study design. However, inter-occasion variability for the hypothetical drug example was small (0.02; 14.2% CV) and the sample size in a cross-over study depends heavily on the magnitude of this variability. A larger variability would therefore result in larger sample sizes for cross-over study designs. Furthermore, type I error rates are inflated for study designs with a small number of patients and/or sparse sampling (*i.e.* the sparsely sampled cross-over study design) (10,11). Sample size calculations for these study designs should therefore be interpreted with caution as the increased percentage of false positives can overestimate the power of a study design.

A cross-over study design requires that the subject is in a similar state at both study occasions. This is often not feasible in the clinical setting particularly if there is a significant acute disease effect. The example of malaria-infected pregnant patients highlights this; there is a continually changing physiological state when malaria is treated which confounds a cross-over comparison. Furthermore, unless there was a second episode of malaria, there is no ethical justification to repeat the drug exposure. For the majority of situations, a parallel comparison will therefore be preferable.

A cross-over study design is potentially less expensive to conduct compared to a parallel design although this depends on complete adherence to the cross-over protocol—and both poor adherence and tracing costs to ensure completion of the study will add to costs.

### Dense *vs* Sparse Sampling

In both the hypothetical drug examples (cross-over and parallel study design) and in the dihydroartemisinin case study, similar or better precisions on all parameter estimates were obtained using the dense sampling design compared to the sparse sampling design. Considering the results obtained with the partly dense and sparse sampling study designs, parameter precision gets generally better with an increasing proportion of densely sampled patients and an increasing number of plasma concentration samples in the study. Regarding the hypothesis testing of the covariate, there is in general more to be gained from including more patients with fewer samples than fewer patients with more samples (10,12). However, a too sparse sampling designs (*e.g.* one sample per individual) in one of the arms of a mixed pharmacokinetic study design can result in lower power because of shrinkage (10). On the other hand, a (partly) densely sampled study design may be preferred because it allows flexible recruitment and so is more likely to be executable in certain circumstances. Fewer patients are required in a (partly) densely sampled study design compared to a study design consisting of solely sparsely sampled individuals. This would be of particular interest for orphan diseases where the patient population to recruit from may be small. There is also a feasibility advantage. In studying acute diseases, it may only be possible to conduct dense sampling on patients admitted in the morning, and then only one at a time, so a pragmatic mix of two sampling strategies may optimise best the opportunities provided.

The required sample size can change as a result of the number of included densely sampled patients, total number of patients, total number of samples and the type of study design (*i.e.* cross-over or parallel design). Furthermore, the effect size (*i.e.* magnitude of covariate effect) and the distribution of the covariate within the study population (*e.g.* 10/90, 20/80, 40/60 or 50/50 distribution for binary covariate in a sample size of 100 patients) can also affect the sample size. Since this study focused on sample size calculations for mixed pharmacokinetic study designs, the impacts of changes in effect size and covariate distribution were not evaluated and values were constant for all evaluated designs.

For certain drugs, optimal sampling schedules for sparse and dense sampling may not be available. In those cases the model structure, the parameters that describe the pharmacokinetics of the drug, the magnitude of covariate effects and the variability parameters should be obtained first from the literature (Fig. 1). An optimal sampling design with reasonably low expected residual errors should then be obtained by making use of optimal design methodology (*e.g.* PopED: http://poped.sourceforge.net/ or PFIM: http://www.pfim.biostat.fr/). Subsequently, simulation-based power calculations can be performed to evaluate the sample sizes for different study designs consisting of part densely and part sparsely sampled patients. This process can be iterative until the outcome is clinically feasible (*i.e.* the sample size is acceptable) and expected residual errors on parameter estimates are acceptable. Combining a power calculation method with optimal design methods for determining the sample sizes and sampling schedules can contribute therefore to a robust and efficient pharmacokinetic study design.

### Economic Aspects

Following a clinical feasibility assessment and an evaluation of the expected optimal precision of parameter estimates, the next step is to consider also the financial costs of a study design. For example, there are no major differences in the precision of parameter estimates for dihydroartemisinin pharmacokinetics between the four proposed study designs, but the study costs vary tremendously.

The difference in study costs in the hypothetical drug example and the selected dihydroartemisinin example is mainly due to the number of samples to be analysed. There is only a small difference in hospitalisation costs as all samples were drawn on the same day (1,000 units for a dense sample study design and 750 units for a sparse sampled study design). However, the hospitalisation costs would have a larger influence on pharmacokinetic study costs when assessing drugs with a long elimination half-life where multiple hospital visits are required for a dense study design compared to fewer hospital visits for a sparse study design.

An alternative approach to compute the overall precision of a model and a certain design is to use the D-criterion (normalised determinant of the observed Fisher information matrix) (13). This enables a comparison of a single measurement value of model precision (D-criterion), from different study designs, to the costs for these designs. However, the applied approach in this research enables the possibility of weighting certain parameters of interest (*e.g.* elimination clearance in case one is less interested in, for example, the absorption phase).

Performing the power calculations and the evaluation of the financial costs of the different study designs can be computationally intensive when complex models and large sample sizes are being evaluated. Nevertheless, the potential cost savings and prevention of doing an under-powered study, by using the studied simulation-based power calculations method, are worth the computational time and cost.

## CONCLUSION

The simulation-based power calculation methodology, based on the Monte Carlo Mapped Power method, enables the successful evaluation of mixed (part sparsely and part densely sampled) study designs. A workflow which guides an easy and straightforward pharmacokinetic study design considering the cost-efficacy of the study was effectively used. This shows that prior knowledge and the evaluated sample size calculation method can contribute significantly the design of a robust and efficient pharmacokinetic study design.
